# Hybrid height and slope figuring method for grazing-incidence reflective optics

**DOI:** 10.1107/S160057752201058X

**Published:** 2023-01-01

**Authors:** Tianyi Wang, Lei Huang, Xiaolong Ke, Yi Zhu, Heejoo Choi, Weslin Pullen, Vipender Negi, Daewook Kim, Mourad Idir

**Affiliations:** a National Synchrotron Light Source II (NSLS-II), Brookhaven National Laboratory, PO Box 5000, Upton, NY 11973, USA; b School of Mechanical and Automotive Engineering, Xiamen University of Technology, Xiamen 361024, People’s Republic of China; c James C. Wyant College of Optical Sciences, University of Arizona, 1630 E. University Blvd, PO Box 210094, Tucson, AZ 85721-0094, USA; d Large Binocular Telescope Observatory, University of Arizona, Tucson, AZ 85721, USA; e Council of Scientific and Industrial Research – Central Scientific Instruments Organisation (CSIR-CSIO), Chandigarh 160030, India; f Academy of Scientific and Innovative Research, Chennai, Tamil Nadu 201002, India; g Department of Astronomy and Steward Observatory, University of Arizona, 933 N. Cherry Avenue, Tucson, AZ 85721, USA; NSRRC, Taiwan

**Keywords:** ion beam figuring, synchrotron optics, two-dimensional, surface slopes

## Abstract

Grazing-incidence reflective optics are commonly used in synchrotron radiation and free-electron laser facilities to transport and focus the emitted X-ray beams. To preserve the imaging capability at the diffraction limit, the fabrication of these optics requires precise control of both the residual height and slope errors. However, all the surface figuring methods are height based, lacking the explicit control of surface slopes. Although our preliminary work demonstrated a one-dimensional (1D) slope-based figuring model, its 2D extension is not straightforward. In this study, a novel 2D slope-based figuring method is proposed, which employs an alternating objective optimization on the slopes in the *x*- and *y*-directions directly. An analytical simulation revealed that the slope-based method achieved smaller residual slope errors than the height-based method, while the height-based method achieved smaller residual height errors than the slope-based method. Therefore, a hybrid height and slope figuring method was proposed to further enable explicit control of both the height and slopes according to the final mirror specifications. An experiment to finish an elliptical-cylindrical mirror using the hybrid method with ion beam figuring was then performed. Both the residual height and slope errors converged below the specified threshold values, which verified the feasibility and effectiveness of the proposed ideas.

## Abstract 2

A novel hybrid height and slope figuring model that enables the explicit control of height and slope errors in synchrotron optics fabrication is described.

## 1. Introduction

With the rapid evolution of free-electron lasers and third- and fourth-generation X-ray synchrotron light sources, the smoothness and precision requirement on X-ray optics have dramatically increased. Grazing-incidence mirrors, a key type of X-ray optics, are widely used for focusing the emitted hard X-ray beams. To preserve the incoming wavefronts and image at the diffraction limit, these mirrors are required to reach the <0.1 µrad root mean square (RMS) level for residual slope errors and <1 nm RMS for residual height errors.

Due to the grazing-incidence geometry, dedicated optical metrology systems have been developed to measure such high-precision mirrors. Initially, slope metrology instruments were developed. The long trace profiler (Takacs *et al.*, 1987 ▸) and the nanometre optical component measuring machine (NOM) (Siewert *et al.*, 2004 ▸) are two well known types of one-dimensional (1D) slope profilers widely used at light source facilities around the world (Qian *et al.*, 1995 ▸; Ali & Yashchuk, 2011 ▸; Siewert *et al.*, 2010 ▸; Nicolas *et al.*, 2016 ▸; Nicolas & Martínez, 2013 ▸; Alcock *et al.*, 2010 ▸). With slightly different configurations from the NOM, the nano-accuracy surface profiler (NSP) was developed (Qian & Idir, 2016 ▸) and advanced (Huang, Wang, Nicolas *et al.*, 2020 ▸; Huang *et al.*, 2022 ▸) using the multi-pitch technique at National Synchrotron Light Source II (NSLS-II), able to reach <0.05 µrad measurement repeatability. Two-dimensional (2D) slope metrology has also been attempted, including the use of the NOM in both the *x-* and *y*-directions (Thiess *et al.*, 2010 ▸), stitching Shack-Hartman (SSH) wavefront sensing (Idir *et al.*, 2014 ▸; Adapa *et al.*, 2021 ▸) and phase measuring deflectometry (Huang, Su *et al.*, 2015 ▸).

As the fourth-generation synchrotron light sources evolve towards almost fully coherent beam, height metrology has become a necessity in the characterization of synchrotron mirrors. Interferometry is the most widely adopted height-measuring technology for mirror characterization. However, since a grazing-incidence mirror is longer in its tangential (*i.e.* *x*) dimension (100 mm to 1 m) than its sagittal (*i.e.* *y*) dimension (5 to 20 mm), stitching interferometry (SI) techniques (Mimura *et al.*, 2005 ▸; Yumoto *et al.*, 2016 ▸; Vivo *et al.*, 2016 ▸; Huang *et al.*, 2019 ▸; Huang, Wang, Tayabaly *et al.*, 2020 ▸) have been developed to extend the field of view. The main idea is to combine the measurements in individual small fields of view to generate a height map of the entire clear aperture (CA) on the mirror surface. The measurement repeatability can reach <0.5 nm RMS for flat and ‘shallow’ curved mirrors (Huang *et al.*, 2019 ▸; Huang, Wang, Tayabaly *et al.*, 2020 ▸). Microscope-based SI techniques (Yamauchi *et al.*, 2003 ▸; Rommeveaux & Barrett, 2010 ▸) have also been attempted to inspect middle- to high-frequency errors.

**Figure 1:**
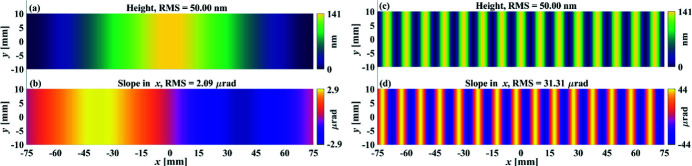
The height errors in (*a*) and (*c*) are generated using equation (1) with (*f*_*x*_, *f*_*y*_) = (150, 0) mm^−1^ and (*f*_*x*_, *f*_*y*_) = (10, 0) mm^−1^, respectively. Although they have the same height error of 50 nm RMS, the corresponding slope errors shown in (*b*) and (*d*) are very different from each other.

On top of the dedicated metrology, deterministic optics fabrication techniques are required to produce grazing-incidence X-ray mirrors. Despite both slope and height metrology instruments being available, the current figuring method in deterministic optics fabrication is height based, in which the height removal is modelled as the convolution between the tool influence function (TIF) of a machine tool and its dwell time at different locations on the mirror surface (Jones, 1977 ▸). Integration from slope to height is thus required if a slope-measuring system is used. It was found that this integration in 1D might amplify the errors if the Riemann approximation was used (Zhou *et al.*, 2016 ▸). Although more advanced integration methods have been proposed to reduce the integration errors (Huang, Idir *et al.*, 2015 ▸; Huang *et al.*, 2017 ▸) and higher-performance height-based optimization algorithms have been developed (Wang, Huang, Kang *et al.*, 2020 ▸; Wang, Huang, Choi *et al.*, 2021 ▸; Wang, Huang, Vescovi *et al.*, 2021 ▸), the control of slope errors is still implicit in the height-based model, which is inappropriate since even two height error maps can give the same r.m.s. residual level; they may correspond to very different slope errors. This is manifested by the sinusoidal surface error to be corrected, *z* ^d^(*x*, *y*), shown in Fig. 1 ▸, defined as

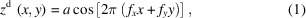

with *a* = 70 nm. The frequencies, (*f*_*x*_, *f*_*y*_), for Figs. 1 ▸(*a*) and 1 ▸(*c*) are (*f*_*x*_, *f*_*y*_) = (150, 0) mm^−1^ and (*f*_*x*_, *f*_*y*_) = (10, 0) mm^−1^, respectively. As shown in Figs. 1 ▸(*b*) and Fig. 1 ▸(*d*), it is obvious that, although the two surface height errors have the same RMS value of 50 nm, the one with a higher *f*_*x*_ corresponds to a larger slope error in the *x* direction. In conventional height-based figuring processes, slope errors were not explicitly monitored and controlled. If there were requirements on residual slope errors, the common solution was to iterate the height-based optimization, usually with smaller machine tools, expecting that the slope would eventually converge with the height. This method is inefficient, and the residual slope error is unpredictable during the optimization process.

Our preliminary research successfully demonstrated a 1D slope-based figuring model (Zhou *et al.*, 2016 ▸), which was further improved by Li & Zhou (2017 ▸). However, the model cannot be simply extended to 2D since one height map corresponds to two slope maps, which are slopes in the *x*- and *y*-directions, respectively, and one dwell time solution should minimize the slope errors in both directions, which is difficult to achieve theoretically. Therefore, in this study, we first formulated the 2D slope-based figuring model and analysed the theoretical difficulty in solving the dwell time from it. Afterwards, an alternating objective optimization method was employed to resolve this difficulty. The analytical simulation indicated that the slope-based optimization achieved smaller residual slope errors than the height-based optimization, while the height-based optimization achieved smaller residual height errors than the slope-based optimization. Based on this constatation, a hybrid height and slope figuring method was proposed, which enables the explicit control of both height and slope errors according to the mirror specifications. Finally, an experiment to finish an elliptical-cylindrical mirror with ion beam figuring (IBF) using the hybrid method was demonstrated. Both the residual height and slope errors converged to the specified values (*i.e.* <1 nm RMS, <0.2 µrad RMS in the *x*-direction, and <0.5 µrad RMS in the *y*-direction), which proved the effectiveness of the proposed ideas.

The rest of the paper is organized as follows. Section 2 formulates the 2D slope-based figuring model, followed by an introduction of the alternating objective optimization used to solve the dwell time from 2D slope errors in Section 3. In Section 4 we explain the hybrid height and slope optimization method and study the performances of the height-based, slope-based and hybrid methods via simulation. The experimental verification is given in Section 5. Section 6 concludes the paper.

## 2. Two-dimensional slope-based figuring model

### 2.1. From height-based to slope-based optimization

The classical 2D height-based figuring model (Jones, 1977 ▸) is defined as

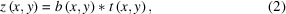

where * represents the convolution operator, and the removed height *z*(*x*, *y*) is modelled as the convolution between the TIF *b*(*x*, *y*) and the dwell time *t*(*x*, *y*). After measuring the desired height removal *z* ^d^ and *b*, the objective is to find a 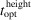 such that

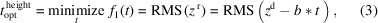

subject to *t* 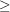 *t*_min_, where *z* ^r^ is the residual errors between *z* ^d^ and *z*, *t*_min_ is the minimum dwell time for each dwell point, and RMS(…) represents the RMS value of ‘…’. According to the property of the convolution, equation (2) can be differentiated as either

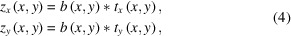

or

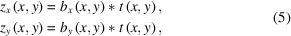

where *g*_*x*_(*x*, *y*) = ∂*g*/∂*x* and *g*_*y*_(*x*, *y*) = ∂*g*/∂*y* represent the slopes of *g*(*x*, *y*) in *x* and *y*, respectively.

Equations (4) and (5) indicate that the differentiations can be applied to either the dwell time or the TIF. Although both of them are mathematically correct, equation (4) is difficult to implement in practice, since it requires both the slope (*i.e.* *z*_*x*_ and *z*_*y*_) and height (*i.e.* *b*) measurements, and the calculated *t*_*x*_ and *t*_*y*_ cannot be directly used. On the other hand, equation (5) enables a pure slope-based process, in which two slope-based equations correspond to one dwell time. Therefore, as an analog to the 1D model (Zhou *et al.*, 2016 ▸; Li & Zhou, 2017 ▸; Zhou *et al.*, 2016 ▸), equation (5) is selected as the 2D slope-based figuring model in this study. After measuring the desired slope removals 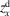 and 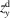 and the TIF slopes *b*_*x*_ and *b*_*y*_, the objectives are to find 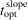 for which

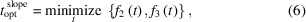

subject to *t* 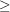 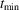 , where

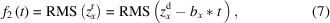

and

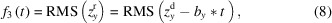

in which 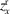 and 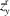 are the residual slope errors in *x* and *y*, respectively.

### 2.2. Difficulty in 2D slope-based optimization

Assuming that the height error to be corrected is defined by the bi-sinusoidal equation shown in equation (1), the corresponding desired slope removal in the *x*- and *y*-directions are

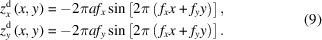

Equation (9) indicates that the slope errors are affected by the frequencies. The higher the values of *f*_*x*_ and *f*_*y*_, the more sensitive the slope errors will correspond to small changes of the amplitude *a*.

The dwell time can be analytically solved from equations (5) and (9). Taking *z*_*x*_(*x*, *y*) as an example, equation (9) can be expressed as the imaginary part of a complex number as

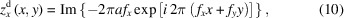

where *i* = 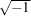 , and Im[…] represents the imaginary part of ‘…’. For convenience, the imaginary operator can be neglected,

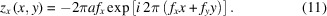

By substituting equation (10) into equation (5) and performing Fourier transforms, equation (5) can be transformed to the frequency domain as

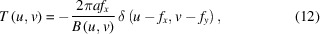

where *T*(*u*, *v*) and *B*(*u*, *v*) are the Fourier transforms of *t*(*x*, *y*) and *b*(*x*, *y*), respectively, and δ(*u*, *v*) is the delta function. Therefore, the dwell time *t*(*x*, *y*) can be solved from equation (12) as

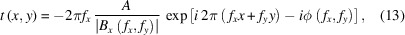

where |*B*_*x*_(*f*_*x*_, *f*_*y*_)| and ϕ(*f*_*x*_, *f*_*y*_) are the amplitude and phase of *B*(*f*_*x*_, *f*_*y*_), respectively. Due to the linear properties of equation (13), the actual dwell time is its imaginary part, which is

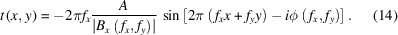

Moreover, to make *t*(*x*, *y*) non-negative, the final *t*(*x*, *y*) should be calculated as

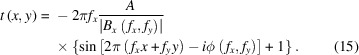

Similarly, the dwell time can be calculated from *z*_*y*_(*x*, *y*) in equations (5) and (9) as

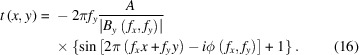

Theoretically, equations (15) and (16) should be satisfied at the same time to ensure that equation (5) is valid. This results in

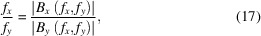

which requires the ratio between each two *f*_*x*_ and *f*_*y*_ to be equal to the ratio between |*B*_*x*_(*f*_*x*_, *f*_*y*_)| and |*B*_*y*_(*f*_*x*_, *f*_*y*_)| at those frequencies. This is extremely difficult to guarantee in practice since a real surface is composed of various *f*_*x*_ and *f*_*y*_.

## 3. 2D slope-based optimization with alternating objectives

Based on the analysis above, we can conclude that optimization by using slope errors is more sensitive to higher frequencies than that using height errors. The slope-based optimization, if it can be solved properly, will thus be more effective in the higher-frequency error ranges, while the height-based optimization is preferred to correct lower-frequency errors. However, due to equation (17), it is theoretically difficult to obtain a reasonable dwell time solution from the two-objective slope-based model. The conventional height-based optimization methods cannot be directly employed. More advanced optimization strategies are thus necessary in the slope-based optimization.

### 3.1. Alternating two-objective optimization

Practically, such a multi-objective optimization problem can only achieve the Pareto optimality (Miettinen, 2012 ▸), at which *t*(*x*, *y*) in equation (6) is optimized in a way that *f*_2_(*t*) cannot improve without *f*_3_(*t*) worsening, and vice versa. Every Pareto optimal point resides on the Pareto front; however, it is non-trivial to find out which point is appropriate for a certain slope-based problem in practice.

The most straightforward way of locating a Pareto-optimal point is the weighting method (Miettinen, 2012 ▸), which combines *f*_2_(*t*) and *f*_3_(*t*) as a single objective, α*f*_2_(*t*) + β*f*_3_(*t*), where α and β are the weights for *f*_2_(*t*) and *f*_3_(*t*), respectively. Nonetheless, it is difficult to determine proper values for α and β. In particular, in a grazing-incidence mirror, the slope errors in the *y*-dimension are more difficult to correct than those in the *x*-dimension, since the *y*-dimension is much shorter than the *x*-dimension due to the grazing-incidence geometry. The obtained dwell time solution thus usually fails to satisfy the specifications on residual slope errors in both *x-* and *y*-dimensions at the same time. To solve this problem, we employed an alternating objective optimization algorithm (see Table S1 of the supporting information), which progressively approaches the specified residual slope errors by iteratively exchanging the search direction between *f*_2_(*t*) and *f*_3_(*t*) using the estimated residual slope errors obtained from the last iteration. Thus, in each step of the alternating objective optimization, only one objective will be optimized so that any of the height-based methods can be used.

In detail, the inputs to the algorithm are specified RMSs of the residual slope errors, 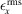 and 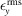 , and the desired slope removals, 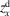 and 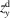 . The algorithm is initialized with a maximum number of iterations, *i*_max_, and the minimum dwell time, *t*_min_, which will be added to each dwell point to enforce 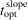 > 0. We use *i*_max_ = 10 in this study, since we found that the algorithm usually converged in less than ten iterations, and *t*_min_ = 0.01 is calculated from the dynamics limits of the translation stages in our IBF system (Wang, Huang, Zhu *et al.*, 2020 ▸). The residual slope errors are initialized with the desired slope removals, 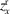 = 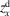 and 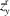 = 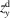 , and the optimized dwell time is initialized as 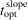 = 0.

Next, the iterations start with the calculation of the current RMSs of the residual slope errors, 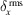 and 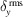 . If both 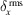 and 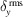 achieve 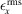 and 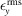 , the current iteration stops and 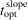 is obtained. Otherwise, the objectives *f*_2_(*t*) and *f*_3_(*t*) are alternatively optimized. In this study, we employed an efficient dwell time optimizer proposed in our previous work (Ke *et al.*, 2022 ▸) to calculate the intermediate dwell time, which is used to update 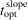 .

Finally, it is crucial to update 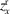 and 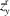 to ensure that the next iteration will be continued on the residual slope errors obtained from the last iteration and thus guarantees the convergence towards 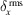 and 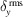 . If the algorithm does not converge in *i*_max_ iterations, it means that the current TIF is not capable of correcting the remaining slope errors. We thus accept 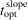 as the solution for the current TIF and select smaller TIFs to repeat the process in pursuit of the eventual convergence to the specifications. With the alternating objective optimization, it is obvious that not only 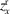 and 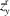 are being minimized simultaneously but also the convergence can be explicitly controlled according to 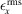 and 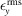 .

## 4. Hybrid height and slope optimization method

In this section, the effectiveness of the alternating objective optimization in the slope-based method is studied. The performances of the height-based and the slope-based methods are compared by applying them to optimize dwell time from analytical surfaces and an analytical Gaussian TIF. First, the sinusoidal surfaces containing the single frequencies shown in Fig. 1 ▸ are tested. Afterwards, the methods are applied to a surface generated with Chebyshev polynomials, which covers a wider range of frequencies. Based on the results, a hybrid height and slope optimization method is then proposed, which enables explicit control of both the residual height and slope errors with respect to the specifications.

### 4.1. Study on the performances of the slope-based and height-based methods

#### 4.1.1. Simulation on single-frequency surfaces

**Figure 2:**
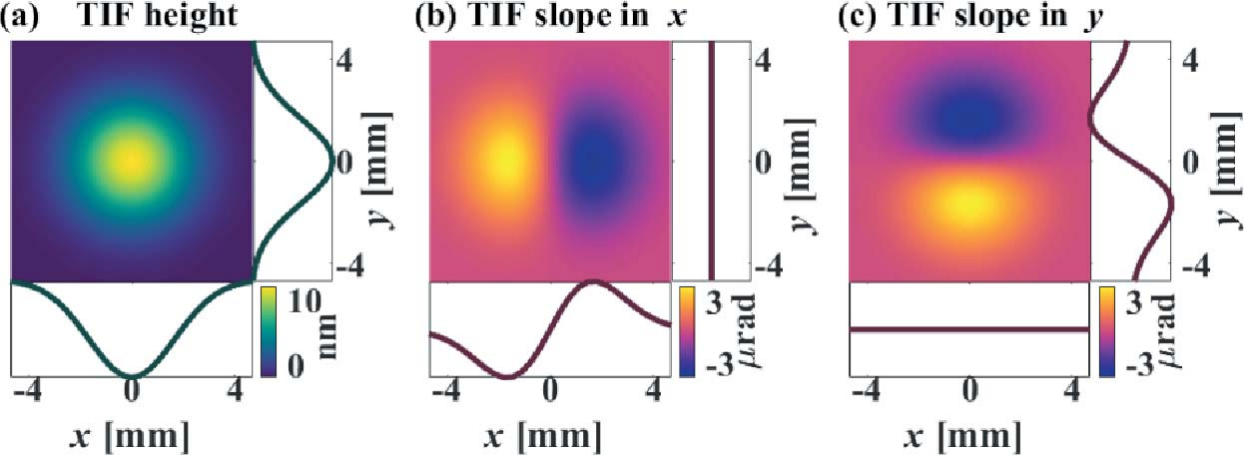
Height (*a*), slope in *x* (*b*) and slope in *y* (*c*) of the analytical Gaussian TIF with FWHM = 5 mm used in the simulation.

The slope-based and height-based methods are first studied on the single-frequency sinusoidal surfaces shown in Fig. 1 ▸ to verify their different performances on low to high frequencies. The employed TIF, as shown in Fig. 2 ▸, is generated by fitting one of our IBF TIFs with a 2D Gaussian function. The diameter of the TIF is 10 mm, with a full width at half-maximum (FWHM) of 5 mm. The dwell grid is thus set as larger than the perimeter of the surfaces by the radius of the TIF, *i.e.* 5 mm. We specify the desired RMSs of the residual height and slope errors as 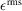 = 1 nm, 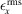 = 0.2 µrad and 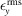 = 0.5 µrad, respectively, which are the same as the specifications imposed in the experiment that will be demonstrated in Section 5.

It is worth mentioning that, to make the comparison fair, both the height-based and slope-based methods use the same optimizer proposed by Ke *et al.* (2022 ▸). Moreover, the height-based method is performed iteratively on the residual height errors until either all the 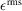 , 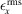 and 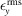 are achieved or *i*_max_ is reached. The final residual height and slope errors are estimated from 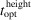 and 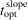 as 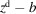 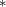 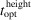 , 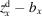 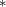 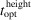 , 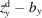 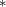 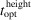 and 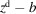 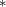 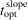 , 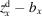 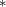 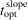 , 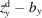 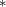 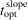 , respectively. These configurations are used in all the simulations demonstrated in this section.

**Figure 3:**
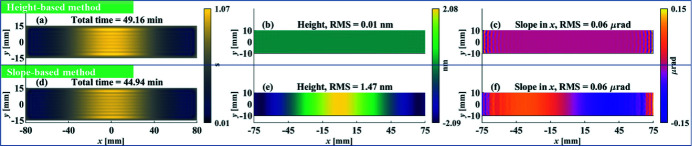
Dwell time, residual height errors and residual slope errors estimated from the height-based method (*a*–*c*) and slope-based method (*d*–*f*), respectively, for the sinusoidal surface shown in Figs. 1 ▸(*a*) and 1 ▸(*b*) with *f*_*x*_ = 150 mm^−1^.

The slope-based and height-based optimizations are first applied to the low-frequency surface error shown in Figs. 1 ▸(*a*) and 1 ▸(*b*), where the period of the error is equal to the length of the mirror. The dwell time, residual height errors and residual slope errors estimated from the height-based and slope-based methods are shown in Figs. 3 ▸(*a*)–3(*c*) and Figs. 3 ▸(*d*)–(*f*), respectively.

It is found that, in this low-frequency example, with the similar total dwell time [Fig. 3 ▸(*a*) and Fig. 3 ▸(*d*)], the height-based method converges to very small residual errors in both the height and slope [Fig. 3 ▸(*b*) and Fig. 3 ▸(*c*)], while the slope-based method could not achieve the specified residual height error [Fig. 3 ▸(*e*)], though the residual slope error converges to the same level [Fig. 3 ▸(*f*)] as that obtained from the height-based method. Therefore, in the low-frequency case, the height-based method outperforms the slope-based method.

**Figure 4:**
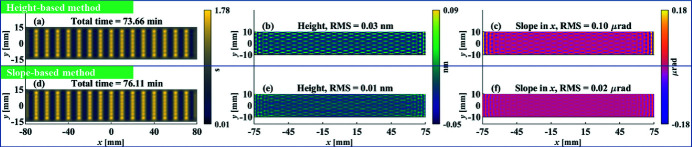
Dwell time, residual height errors and residual slope errors estimated from the height-based method (*a*–*c*) and slope-based method (*d*–*f*), respectively, for the sinusoidal surface shown in Figs. 1 ▸(*c*) and 1 ▸(*d*) with *f*_*x*_ = 10 mm^−1^.

The same operations are then applied to the higher-frequency surface error shown in Figs. 1 ▸(*c*) and 1 ▸(*d*), in which one period of the surface error is equal to the diameter of the TIF. The dwell time, residual height errors and residual slope errors estimated from the height-based and slope-based methods are shown in Figs. 4 ▸(*a*)–4(*c*) and Figs. 4 ▸(*d*)–4(*f*), respectively.

It is obvious that, with similar total dwell time [Figs. 4 ▸(*a*) and 4 ▸(*d*)], as the frequency increases, the slope-based method converges to smaller residual errors in both the height and slope [Figs. 4 ▸(*e*) and 4 ▸(*f*)] than those obtained from the height-based method [Figs. 4 ▸(*b*) and 4 ▸(*c*)], which indicates that the slope-based method is preferred when correction of relatively high-frequency errors is necessary.

#### 4.1.2. Simulation on a multi-frequency surface

**Figure 5:**
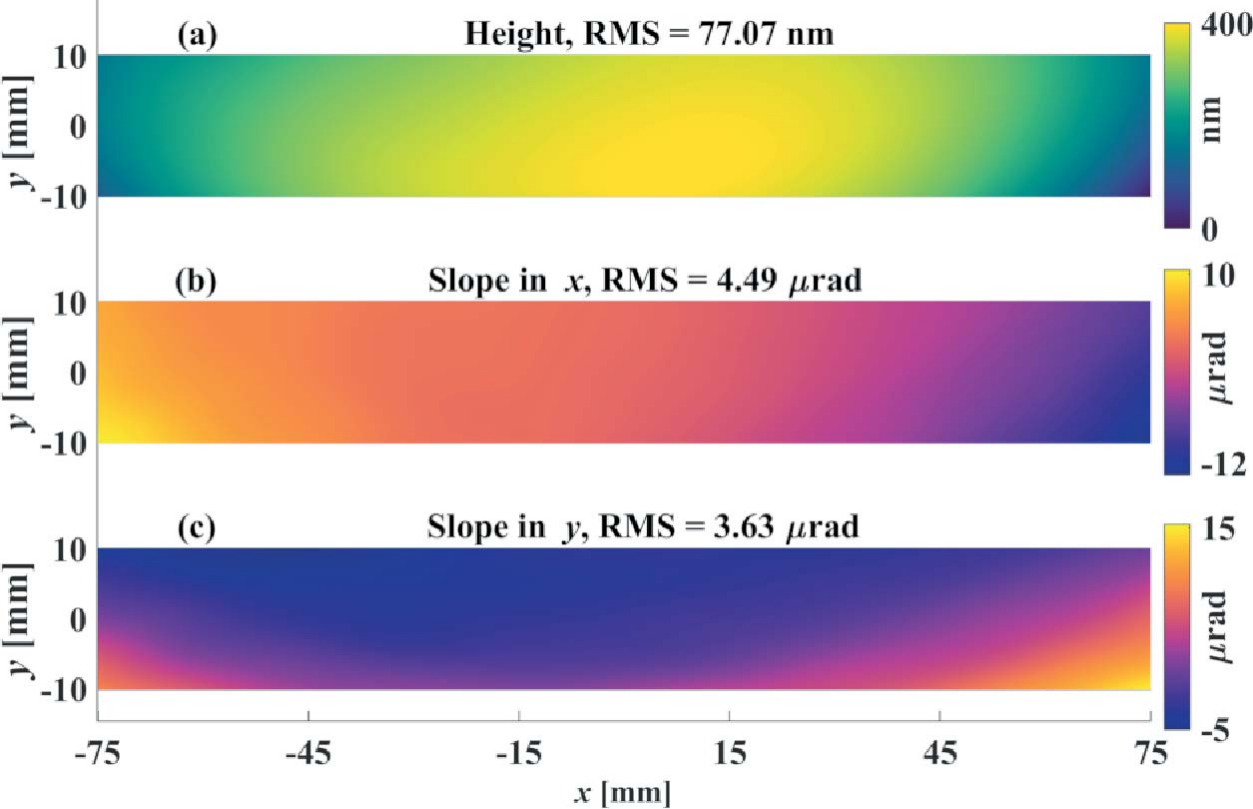
Height (*a*), slope in *x* (*b*) and slope in *y* (*c*) of an analytical surface generated with a 260-order Chebyshev polynomial.

Based on the single-frequency simulation results above, it is clear that the height-based and slope-based methods have different frequency responses. To futher understand the performance of the slope-based and height-based methods on real surfaces, which are always composed of various low-to-high-frequency components, they are applied to the surface height and slopes shown in Fig. 5 ▸.

The surface height [Fig. 5 ▸(*a*)] and slope errors [Figs. 5 ▸(*b*) and 5 ▸(*c*)] are generated by fitting a measured surface error map with a 10 × 26 = 260-order Chebyshev polynomial. The clear aperture of the surface is 20 mm × 150 mm and the lateral resolution is 0.1 mm, which is the same as for our SI system (Huang, Wang, Tayabaly *et al.*, 2020 ▸). The initial RMSs of *z* ^d^, 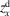 and 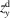 are 77.0 nm, 4.49 µrad and 3.63 µrad, respectively, which are close to the measurements.

**Figure 6:**
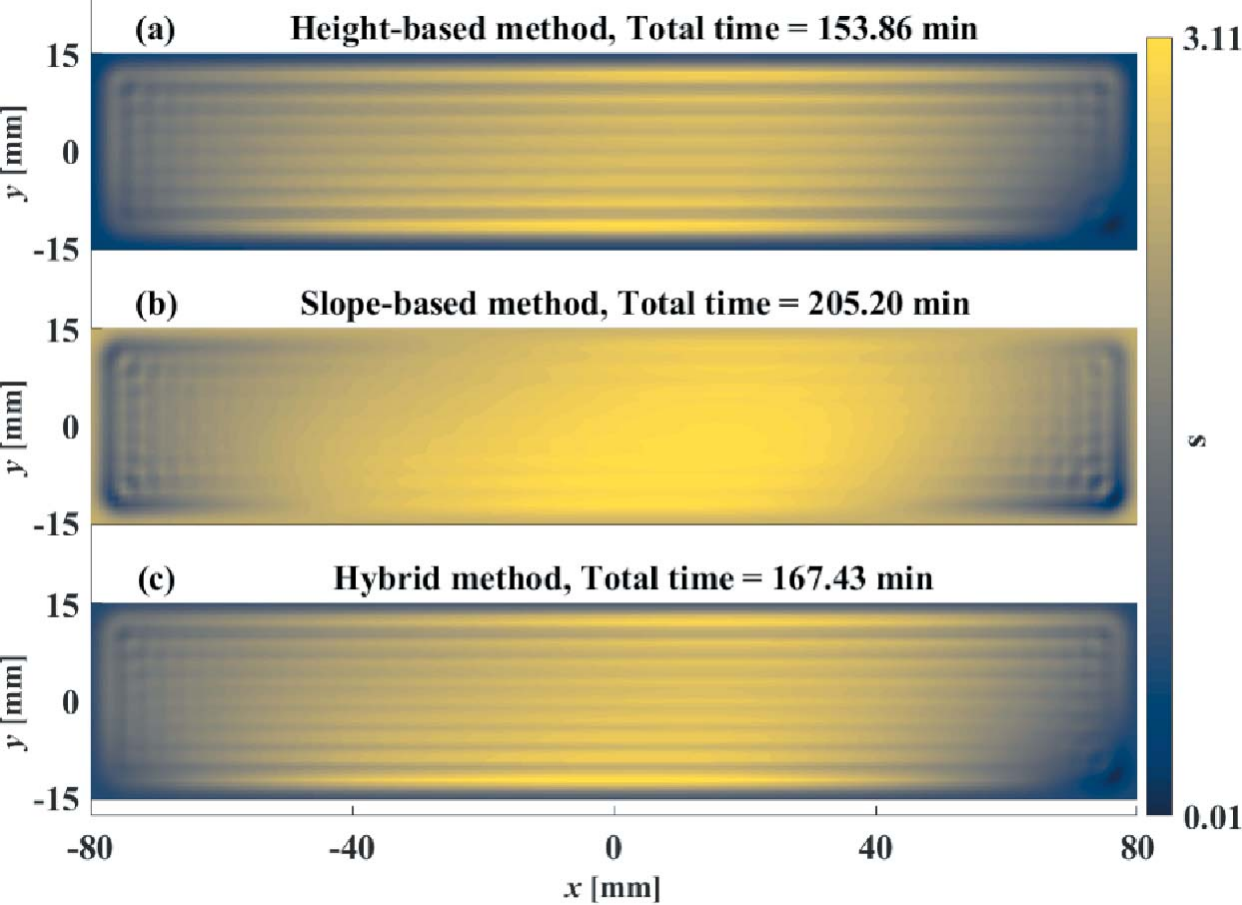
Dwell time optimized from the height-based method (*a*), slope-based method (*b*) and hybrid method (*c*).

**Figure 7:**
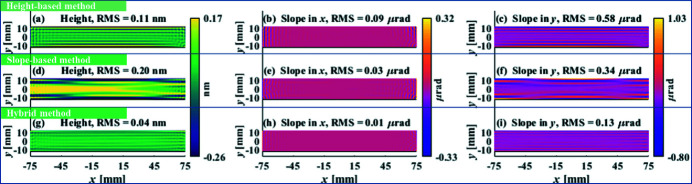
Residual height and slope errors estimated from the height-based method (*a*–*c*), slope-based method (*d*–*f*) and hybrid method (*g*–*i*).

The optimized 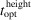 and 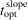 are demonstrated in Figs. 6 ▸(*a*) and 6 ▸(*b*), respectively, from which the corresponding residual height and slopes errors are estimated in Figs. 7 ▸(*a*)–7(*c*) and Figs. 7 ▸(*d*)–7(*f*), respectively. In terms of the residual height errors, the height-based method, as shown in Figs. 6 ▸(*a*) and 7 ▸(*a*), outperforms the slope-based method shown in Figs. 6 ▸(*b*) and 7 ▸(*d*). Although both the height-based and slope-based methods reach the specified 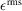 , the height-based method achieves a smaller (about a factor of 0.5) residual height error in a shorter (51 min less) total dwell time.

However, as shown in Fig. 7 ▸(*c*), the height-based method fails to achieve the specified 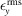 , which means that the height-based method does not converge in *i*_max_ iterations. On the other hand, the slope-based method, as shown in Figs. 7 ▸(*e*) and 7 ▸(*f*), reaches both the specified 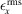 and 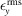 in only three iterations. Moreover, the achieved residual slope error in the *x*-dimension is two times smaller than that obtained from the height-based method shown in Fig. 7 ▸(*b*). In a word, the height-based method is better at reducing the height errors while the slope-based method is preferred when the residual slope errors are not within the specifications.

**Figure 8:**
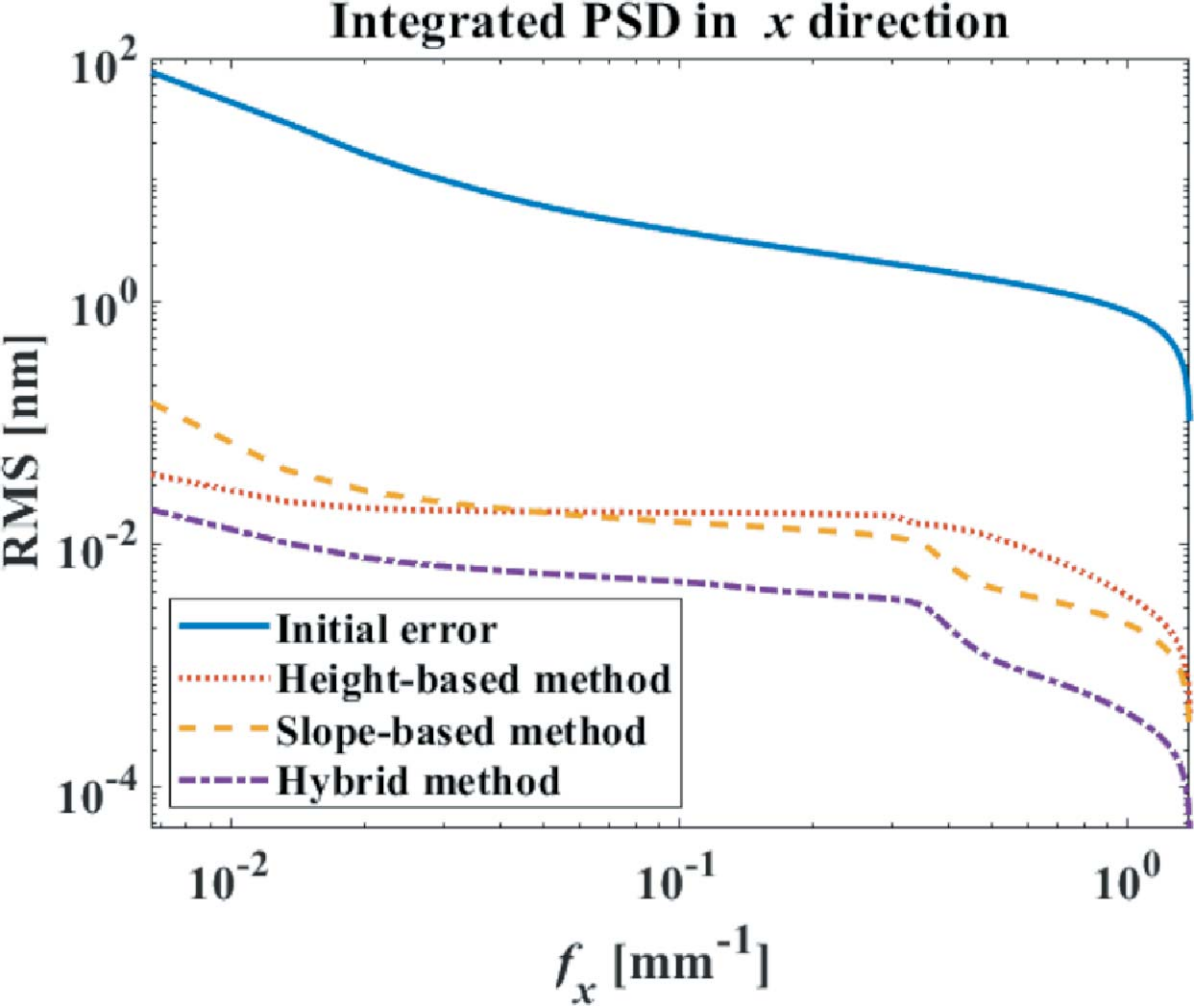
Integrated PSD distributions in the *x*-direction calculated from the residual height errors estimated with the height-based, slope-based and hybrid methods.

From the exploitation of the integrated power spectral density (PSD) distributions of the residual height errors shown in Fig. 8 ▸, it is found that the height-based method achieves a lower PSD in the low-frequency range while the slope-based method is better at the middle-frequency range, which reveals the different sensitivities of height and slope to the different spatial frequencies of errors.

### 4.2. Alternating three-objective optimization

The result shown in Section 4.1 demonstrates that both 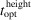 and 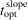 are reasonable dwell time solutions for their respective height-based or slope-based objectives. However, they may fail to minimize the other objectives that are not included in the optimization process. In other words, minimization of *f*_1_(*t*) does not guarantee the minimization of *f*_2_(*t*) or *f*_3_(*t*), and vice versa. Therefore, if there are specifications on both the residual height and slope errors, it is more appropriate to define the entire optimization problem as

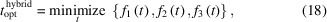

subject to *t* 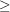 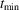 , which contains all the three objectives and should offer a more universal solution that may integrate the merits of both the height-based and slope-based optimizations. Therefore, we propose a hybrid height and slope method, which extends the idea of the alternating two-objective optimization (see Table S1) by including the height-based optimization in the iterative process (see Table S2).

To achieve faster convergence, two key differences from the alternating two-objective optimization are worth emphasizing here. First, at the end of each objective optimization, both the residual height and slope errors will be estimated using the current 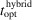 , no matter whether it is obtained from the height-based or the slope-based process. This ensures that *z* ^r^, 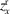 and 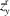 are minimized in the current iteration. Second, the height-based process (lines 7–12 in Table S2) is first performed in each iteration, since slope errors will always be reduced with a decrease in the height errors.

The performance of the hybrid method is compared with the height-based and slope-based methods. As shown in Fig. 6 ▸(*c*), after three iterations the hybrid method takes a little longer (14 min more) total dwell time than the height-based method. However, as shown in Figs. 7 ▸(*a*), 7 ▸(*b*) and 7 ▸(*c*), both the residual height and slope errors greatly outperform those obtained from the height-based and slope-based methods. By further examining the PSD distribution in Fig. 8 ▸, the hybrid method is also found to be superior to both the height-based and slope-based methods over the entire spatial frequency range.

All these simulation results suggest that either the height-based or the slope-based method underestimates the removal capability of a TIF, while the hybrid method achieves a more universal dwell time solution. It not only guarantees a more rapid convergence towards the specifications but also enables explicit control of the optimization based on the specifications. It indicates that the hybrid method is necessary and effective in dwell time optimization when there are specifications on height and slope simultaneously.

## 5. Experiment

To further verify the feasibility of the hybrid method in practice, we applied it to finish one of our grazing-incidence mirrors. Recently, we received a request from the In situ and Operando Soft X-ray Spectroscopy (IOS) beamline at NSLS-II to produce a silicon horizontal Kirkpatrick–Baez (KB) mirror using our IBF system (Wang, Huang, Zhu *et al.*, 2020 ▸). We thus had the opportunity to test our IBF solutions. The specifications for the elliptical-cylindrical KB mirror are shown in Table 1 ▸.

**Figure 9:**
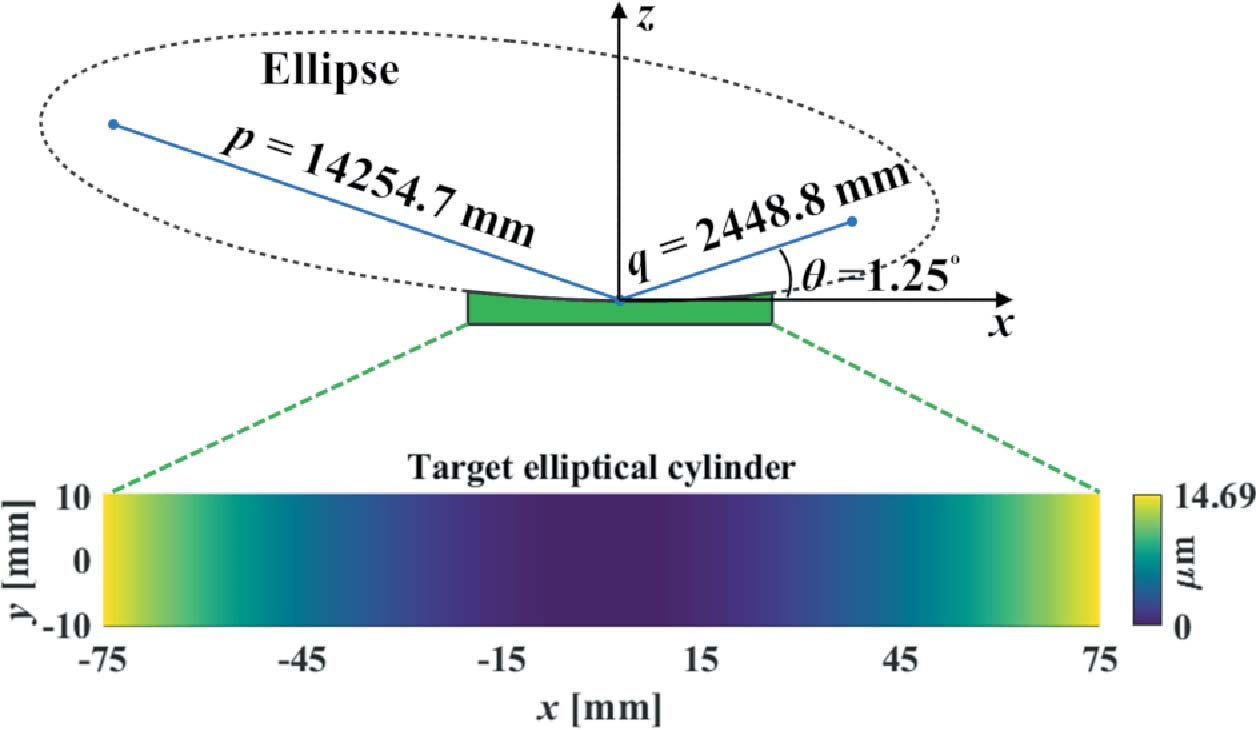
Target elliptical-cylindrical mirror with the object distance *p* = 14254.7 mm, image distance *q* = 2448.8 mm and grazing angle θ = 1.25°.

The size of the CA is 20 mm × 150 mm. The object distance, image distance and grazing angle of the off-axis ellipse, as schematically shown in Fig. 9 ▸, are *p* = 14254.7 mm, *q* = 2448.8 mm and θ = 1.25°, respectively. The height, tangential slope and sagittal slope errors from the target shape should be less than 1 nm RMS, 0.2 µrad RMS and 0.5 µrad RMS, respectively, and the surface micro-roughness should not exceed 0.3 nm RMS.

### 5.1. Experimental setup

**Figure 10:**
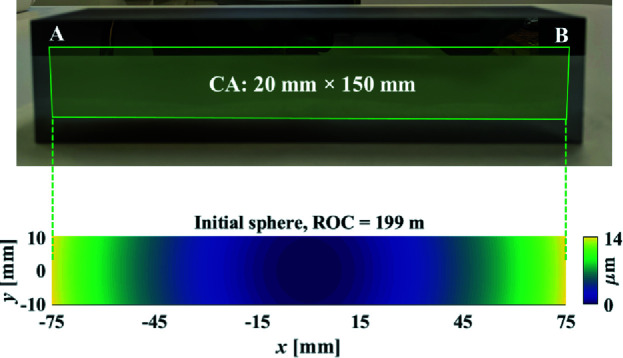
Initial spherical mirror with ROC = 199 m.

To minimize the processing time, we started by finding the best-fit sphere to the target elliptical cylinder. We fit the ellipse parameters in Table 1 ▸ to a circle and found that the optimal radius of curvature (ROC) of the circle that achieved the minimal average material removal is 199 m. Therefore, as shown in Fig. 10 ▸, we purchased a 30 mm × 160 mm spherical mirror, which had been shaped to be within ±1% of the expected ROC, as the base mirror. Also, the mirror was further pitch-polished to achieve the specified roughness level, since IBF can hardly reduce but introduce minimal damage to the roughness (Mikhailenko *et al.*, 2022 ▸). The left and right ends of the mirror were marked as A and B, respectively.

**Figure 11:**
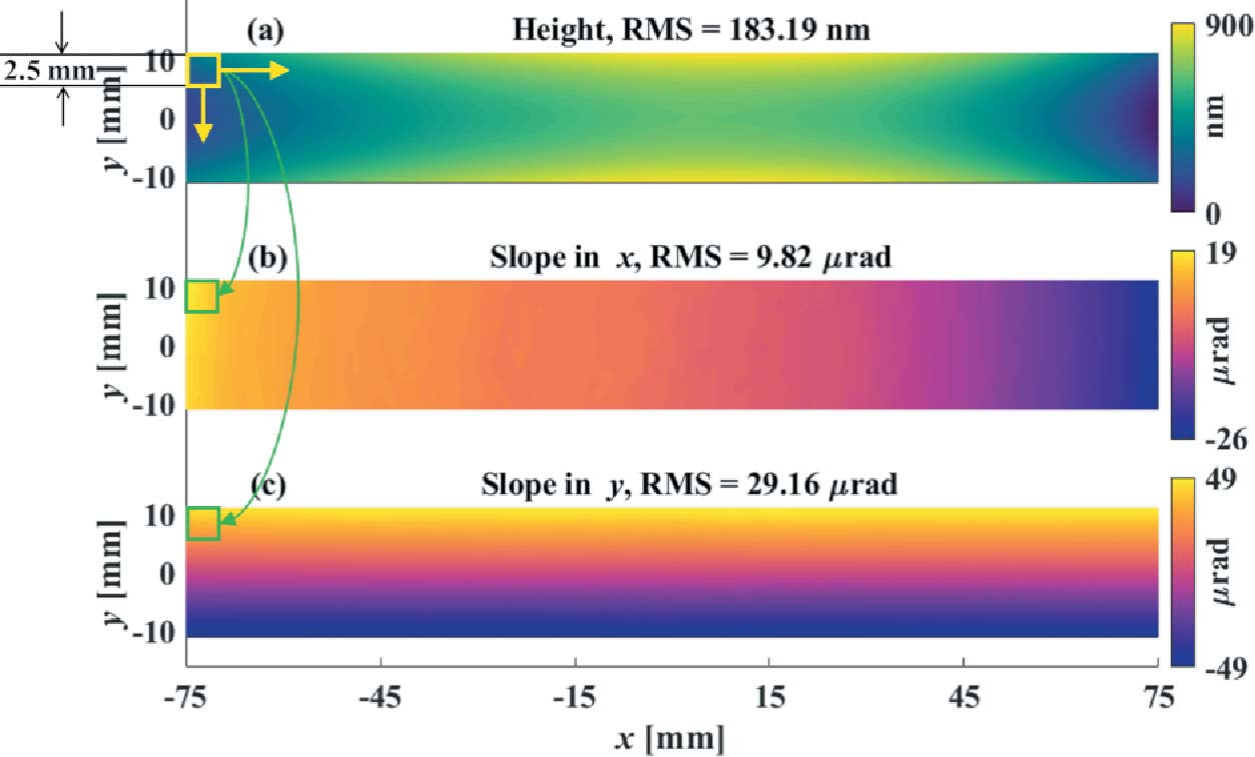
Desired removals of height (*a*), slope in *x* (*b*) and slope in *y* (*c*) from the sphere to the elliptical cylinder, where the slope maps are generated from the height map with the 2.5 mm sliding window.

The desired height removal from the initial spherical mirror to the target elliptical-cylindrical mirror is shown in Fig. 11 ▸(*a*), where the initial height error is 183.19 nm RMS. In this experiment, as an example shown in Figs. 11 ▸(*b*) and 11 ▸(*c*), the 2D slope data were generated from the height measurement using a 2.5 mm × 2.5 mm sliding window in the experiment. We found that this method excellently matches the 2.5 mm pinhole used in our NSLS-II NSP system (Huang, Wang, Tayabaly *et al.*, 2020 ▸). This will be further manifested in the final inspection cross-validated between our SI and NSP systems in Section 5.4.

**Figure 12:**
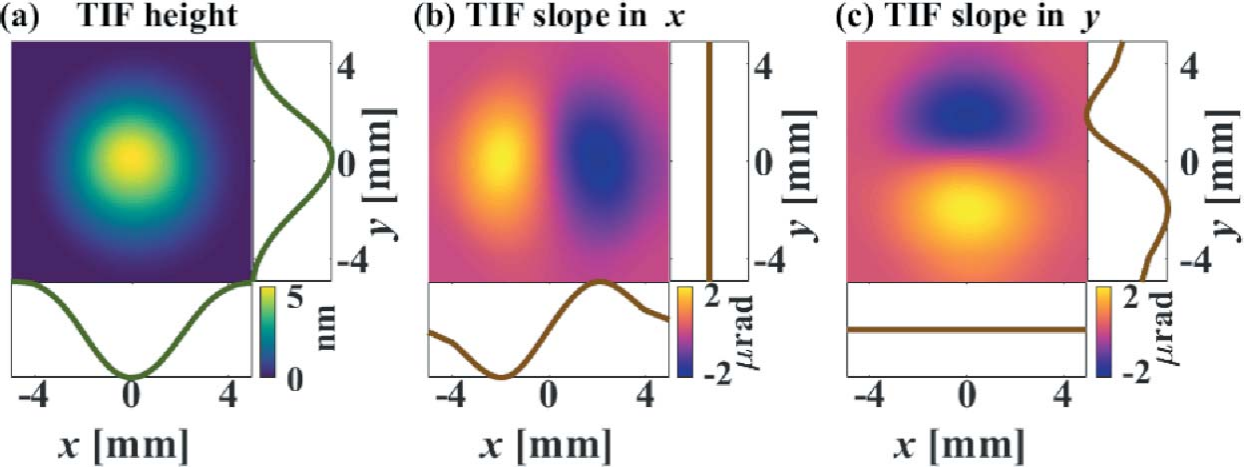
Height (*a*), slope in *x* (*b*) and slope in *y* (*c*) of the IBF TIF generated with a 5 mm diaphragm.

The IBF system (see Fig. S1 of the supporting information) is equipped with a KDC10 gridded ion source from Kaufman & Robinson Inc. The working parameters for the ion source are beam voltage *U*_b_ = 600 V, beam current *I*_b_ = 10 mA, accelerator voltage *U*_a_ = −90 V and accelerator current *I*_a_ = 2 A. One of the most frequently used TIFs of the IBF system is shown in Fig. 12 ▸, which is obtained by placing a 5 mm diaphragm in front of the ion source to constrain the shape of the ion beam. The radius of the TIF is 5 mm, with the FWHM equal to 4.4 mm. The peak removal rate is 5.5 nm s^−1^ and the volumetric removal rate is 98.8 nm mm^2^ s^−1^.

### 5.2. Height-based figuring of the mirror

**Figure 13:**
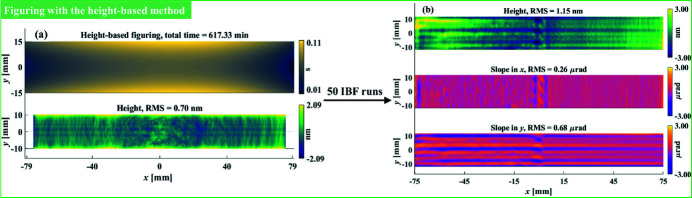
The initial figuring was guided by the estimation obtained from the height-based method (*a*). The figuring result after 50 IBF runs was measured with the SI system (*b*).

Initially, before we proposed the hybrid method, we started the IBF of the mirror based on the desired height removal and TIF shown in Figs. 11 ▸(*a*) and 12 ▸(*a*), respectively. The height-based estimation is given in Fig. 13 ▸(*a*), which used a raster tool path with 0.5 mm machining intervals. The total processing time is more than ten hours, which is too long to be completed in a single IBF run. Therefore, to maintain the stability of the ion beam and reduce the non-linearity in material removal caused by the thermal effect, we divided the dwell time into 50 IBF runs before sending to our IBF system. We monitored the intermediate residual height errors by measuring the mirror every ten cycles. The measured residual height error map after the 50 cycles of IBF is shown in Fig. 13 ▸(*b*), from which the residual slope errors in *x* and *y* were calculated from the residual height errors using the method mentioned in Section 5.1 and Fig. 11 ▸.

It is worth mentioning that the small ‘bumps’ shown in Fig. 13 ▸(*b*) resulted from the stage failure during the tenth cycle. However, since the damage was rather small compared with the total removal amount, we thus continued the experiment to see whether the mirror could still meet the specification with these artefacts. As shown in Fig. 13 ▸(*b*), it was found that both the residual height and slope errors (*i.e.* 1.15 nm RMS, 0.26 µrad RMS and 0.68 µrad RMS, respectively) were still larger than the specifications given in Table 1 ▸. Conventionally, to further pursue the specifications, we would repeat the height-based IBF process with a smaller TIF, expecting that the slope will finally converge with the height. However, in this study, to testify the feasibility of the proposed hybrid method, we tried to apply it to finish the mirror using the same TIF shown in Fig. 12 ▸.

### 5.3. Finishing of the mirror with the hybrid method

**Figure 14:**
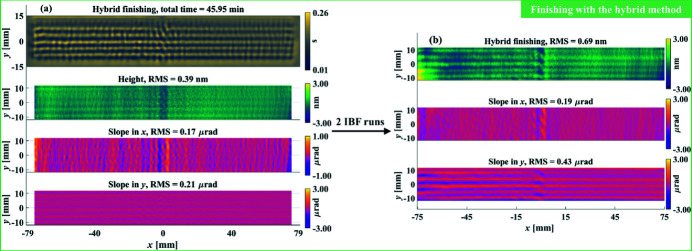
The finishing was guided by the estimation obtained from the hybrid method (*a*). The finishing result after two IBF runs was measured with the SI system (*b*).

The estimations obtained from the hybrid method based on the measurement in Fig. 13 ▸(*b*) are shown in Fig. 14 ▸(*a*), which shows that both the height and slope specifications can be achieved in 45.95 min. Similar to the figuring process, the dwell time was divided into two IBF runs, after which the final residual height and slope errors shown in Fig. 14 ▸(*b*) reached 0.69 nm RMS, 0.19 µrad RMS and 0.43 µrad RMS, respectively. Although the measured residual errors were slightly larger than the estimations due to the actual hardware limits, they all achieved the specifications, and the final height and slope convergence ratios are 99.6%, 98.1% and 98.5%, respectively, which verifies the feasibility of the proposed hybrid height and slope optimization method and the efficiency of our IBF system.

It is worth reiterating that the estimations in the real experiment shown in Figs. 13 ▸ and 14 ▸ are worse than those of the simulation given in Fig. 7 ▸ within an order of magnitude. This is due to the unavoidable noise from either the metrology instruments or the IBF processes. From the metrology, our SI and NSP are within the high-frequency uncorrectable noise levels of 0.3 nm and 50 nrad, respectively. During an IBF process, positioning errors, thermal effects and dynamic limits also contribute to the overall uncertainties.

### 5.4. Final inspection of the mirror

**Figure 15:**
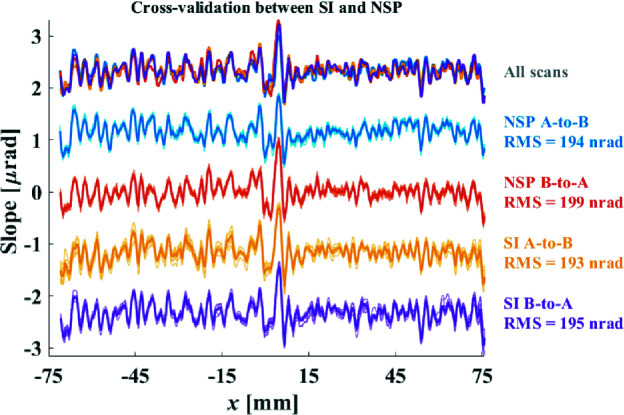
Cross-validation between the SI and NSP measurements of the residual slope errors along the *x*-direction.

To confirm that the finished mirror had achieved all the specifications given in Table 1 ▸, the final inspection was performed using the NSP (Huang, Wang, Nicolas *et al.*, 2020 ▸) for slope measurement, the SI for height measurement and a Zygo NewView white-light interferometer for roughness examination. Each NSP or SI measurement was performed ten times from A to B then B to A. The residual height errors measured with the SI system achieved the same RMS level as Fig. 14 ▸(*b*); however, to guarantee that the height measurements are reliable, we decided to cross-validate them using our NSP system. As the NSP is a 1D slope profiler, we inspected the centre line of the mirror along the *x*-direction. The centre lines from the SI measurements were also extracted and converted into slope profiles using the method mentioned in Section 5.1. Fig. 15 ▸ demonstrates the slope profiles of the centre line of the mirror measured with both the NSP and SI, with the average of every ten scans highlighted in bold. It was found that the measurements obtained from two different instruments validated each other and the residual slope errors were all below the specifications, which proved that the fabricated mirror had achieved both the residual height and slope specifications (refer to Figs. S2 and S3 for more details of the inspection reports).

## 6. Conclusion

In this study, we proposed a two-dimensional slope-based figuring model, enabling the dwell time to be directly optimized using slope data from measurements. Due to the two objective functions in the slope-based model, we introduced an alternating optimization algorithm to iteratively approach both objectives. From the comparison between the height-based and slope-based methods, we found that the height-based method is better at reducing residual height errors while the slope-based method is preferred when there are strict requirements on residual slope errors. Based on this constatation, we proposed the hybrid height and slope-based optimization method which alternatively minimizes both the height and slope objectives. From the simulation result, it was found that the hybrid method outperformed both the height-based and slope-based methods in the entire range of spatial frequencies, which is especially useful for synchrotron mirrors which have strict specifications on residual height and slope errors simultaneously. Finally, we applied the hybrid method to finish an elliptical-cylindrical mirror using ion beam figuring. The mirror has achieved all the specifications, which proves the effectiveness of the proposed ideas. We thus recommend using this method for synchrotron mirror fabrication.

## Supplementary Material

Tables S1 and S2; Figures S1 to S3. DOI: 10.1107/S160057752201058X/ju5048sup1.pdf

## Figures and Tables

**Table 1:** Specifications of the elliptical-cylindrical mirror

CA (mm)	*p* (mm)	*q* (mm)	θ (°)	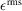 (nm)	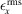 (µrad)	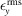 (µrad)	Roughness (nm)
20 × 150	14254.7	2448.8	1.25	<1	<0.2	<0.5	≤0.3

## References

[bb1] Adapa, B. R., Dovillaire, G., Vivo, A., Perrin, F., Mayer, R. & Barrett, R. (2021). *Rev. Sci. Instrum.* **92**, 113103. 10.1063/5.0067871 34852555

[bb2] Alcock, S., Sawhney, K., Scott, S., Pedersen, U., Walton, R., Siewert, F., Zeschke, T., Senf, F., Noll, T. & Lammert, H. (2010). *Nucl. Instrum. Methods Phys. Res. A*, **616**, 224–228.

[bb3] Ali, Z. & Yashchuk, V. V. (2011). Report LBNL-5587E. Lawrence Berkeley National Lab.oratory, Berkeley, CA, USA.

[bb4] Huang, L., Idir, M., Zuo, C., Kaznatcheev, K., Zhou, L. & Asundi, A. (2015). *Opt. Lasers Eng.* **64**, 1–11.

[bb5] Huang, L., Wang, T., Nicolas, J., Polack, F., Zuo, C., Nakhoda, K. & Idir, M. (2020). *Opt. Express*, **28**, 23060–23074. 10.1364/OE.392433 32752308

[bb6] Huang, L., Wang, T., Nicolas, J., Vivo, A., Polack, F., Thomasset, M., Zuo, C., Tayabaly, K., Wook Kim, D. & Idir, M. (2019). *Opt. Express*, **27**, 26940–26956. 10.1364/OE.27.026940 31674564

[bb7] Huang, L., Wang, T., Polack, F., Nicolas, J., Nakhoda, K. & Idir, M. (2022). *Front. Phys.* **10**, 880772.

[bb8] Huang, L., Wang, T., Tayabaly, K., Kuhne, D., Xu, W., Xu, W., Vescovi, M. & Idir, M. (2020). *Opt. Lasers Eng.* **124**, 105795.

[bb9] Huang, L., Xue, J., Gao, B., Zuo, C. & Idir, M. (2017). *Opt. Lasers Eng.* **91**, 221–226.

[bb10] Huang, R., Su, P., Burge, J. H., Huang, L. & Idir, M. (2015). *Opt. Eng.* **54**, 084103.

[bb11] Idir, M., Kaznatcheev, K., Dovillaire, G., Legrand, J. & Rungsawang, R. (2014). *Opt. Express*, **22**, 2770–2781. 10.1364/OE.22.002770 24663568

[bb12] Jones, R. A. (1977). *Appl. Opt.* **16**, 218–224. 10.1364/AO.16.000218 20168455

[bb13] Ke, X., Wang, T., Zhang, Z., Huang, L., Wang, C., Negi, V. S., Pullen, W. C., Choi, H., Kim, D. & Idir, M. (2022). *Opt. Express*, **30**, 16957–16972. 10.1364/OE.456855 36221529

[bb14] Li, Y. & Zhou, L. (2017). *Proc. SPIE*, **10460**, 104601X.

[bb15] Miettinen, K. (2012). *Nonlinear Multiobjective Optimization.* Springer Science & Business Media.

[bb16] Mikhailenko, M., Pestov, A., Chkhalo, N., Zorina, M., Chernyshev, A., Salashchenko, N. & Kuznetsov, I. (2022). *Appl. Opt.* **61**, 2825–2833. 10.1364/AO.455096 35471358

[bb17] Mimura, H., Yumoto, H., Matsuyama, S., Yamamura, K., Sano, Y., Ueno, K., Endo, K., Mori, Y., Yabashi, M., Tamasaku, K., Nishino, Y., Ishikawa, T. & Yamauchi, K. (2005). *Rev. Sci. Instrum.* **76**, 045102.

[bb18] Nicolas, J. & Martínez, J. C. (2013). *Nucl. Instrum. Methods Phys. Res. A*, **710**, 24–30.

[bb19] Nicolas, J., Pedreira, P., Šics, I., Ramírez, C. & Campos, J. (2016). *Proc. SPIE*, **9962**, 996203.

[bb20] Qian, S. & Idir, M. (2016). *Proc. SPIE*, **9687**, 96870D.

[bb21] Qian, S., Jark, W. & Takacs, P. Z. (1995). *Rev. Sci. Instrum.* **66**, 2562–2569.

[bb22] Rommeveaux, A. & Barrett, R. (2010). *Nucl. Instrum. Methods Phys. Res. A*, **616**, 183–187.

[bb23] Siewert, F., Buchheim, J. & Zeschke, T. (2010). *Nucl. Instrum. Methods Phys. Res. A*, **616**, 119–127.

[bb24] Siewert, F., Noll, T., Schlegel, T., Zeschke, T. & Lammert, H. (2004). *AIP Conf. Proc.* **705**, 847–850.

[bb25] Takacs, P. Z., Qian, S. & Colbert, J. (1987). *Proc. SPIE*, **0749**, 59–64.

[bb26] Thiess, H., Lasser, H. & Siewert, F. (2010). *Nucl. Instrum. Methods Phys. Res. A*, **616**, 157–161.

[bb27] Vivo, A., Lantelme, B., Baker, R. & Barrett, R. (2016). *Rev. Sci. Instrum.* **87**, 051908. 10.1063/1.4950745 27250380

[bb28] Wang, T., Huang, L., Choi, H., Vescovi, M., Kuhne, D., Zhu, Y., Pullen, W. C., Ke, X., Kim, D. W., Kemao, Q., Tayabaly, K., Bouet, N. & Idir, M. (2021). *Opt. Express*, **29**, 15114–15132. 10.1364/OE.419490 33985218

[bb29] Wang, T., Huang, L., Kang, H., Choi, H., Kim, D. W., Tayabaly, K. & Idir, M. (2020). *Sci. Rep.* **10**, 8135. 10.1038/s41598-020-64923-3 PMC7235036 32424222

[bb30] Wang, T., Huang, L., Vescovi, M., Kuhne, D., Zhu, Y., Negi, V. S., Zhang, Z., Wang, C., Ke, X., Choi, H., Pullen, W. C., Kim, D., Kemao, Q., Nakhoda, K., Bouet, N. & Idir, M. (2021). *Opt. Express*, **29**, 38737. 10.1364/OE.443346 34808920

[bb31] Wang, T., Huang, L., Zhu, Y., Vescovi, M., Khune, D., Kang, H., Choi, H., Kim, D. W., Tayabaly, K., Bouet, N. & Idir, M. (2020). *Appl. Opt.* **59**, 3306–3314. 10.1364/AO.389010 32400440

[bb32] Yamauchi, K., Yamamura, K., Mimura, H., Sano, Y., Saito, A., Ueno, K., Endo, K., Souvorov, A., Yabashi, M., Tamasaku, K., Ishikawa, T. & Mori, Y. (2003). *Rev. Sci. Instrum.* **74**, 2894–2898.

[bb33] Yumoto, H., Koyama, T., Matsuyama, S., Yamauchi, K. & Ohashi, H. (2016). *Rev. Sci. Instrum.* **87**, 051905. 10.1063/1.4950714 27250377

[bb34] Zhou, L., Huang, L., Bouet, N., Kaznatcheev, K., Vescovi, M., Dai, Y., Li, S. & Idir, M. (2016). *J. Synchrotron Rad.* **23**, 1087–1090. 10.1107/S1600577516010882 27577760

