# Supplementary material for: Hybrid height and slope figuring method for grazing-incidence reflective optics
Source: J Synchrotron Radiat. 2023 Jan 1;30(Pt 1):65–75. doi: 10.1107/S160057752201058X (PMC9814051; doi:10.1107/S160057752201058X)
Supplement: Supplementary file 1 [file s-30-00065-sup1.pdf]

JOURNAL OF SYNCHROTRON RADIATION

Volume 30 (2023)

Supporting information for article:

Hybrid height and slope figuring method for grazing-incidence reflective optics

Tianyi Wang, Lei Huang, Xiaolong Ke, Yi Zhu, Heejoo Choi, Weslin Pullen, Vipender Negi, Daewook Kim and Mourad Idir

Table S1 Alternating 2-objective optimization for the slope-based method.

Input: ϵ_x^{rms}, ϵ_y^{rms}, z_x^d, z_y^d

Output: t_{opt}^{slope}

Initialization: i_{max}, t_{min}, $z_x^r = z_x^d$, $z_y^r = z_y^d$, $t_{opt}^{slope} = 0$;

while $i = 1$ **to** i_{max} **do**

    $\delta_x^{rms} = \mathrm{RMS}(z_x^r)$, $\delta_y^{rms} = \mathrm{RMS}(z_y^r)$;

    if $\delta_x^{rms} \le \epsilon_x^{rms}$ **and** $\delta_y^{rms} \le \epsilon_y^{rms}$

        break;

    else

        if $\delta_x^{rms} > \epsilon_x^{rms}$

            $t_2 = \mathrm{Optimize\_}f_2(z_x^r, b_x)$;

            $t_{opt}^{slope} = t_{opt}^{slope} + t_2$;

            $t_{opt}^{slope} = t_{opt}^{slope} - \min(t_{opt}^{slope}) + t_{min}$;

            $z_x^r = z_x^d - b_x * t_{opt}^{slope}$, $z_y^r = z_y^d - b_y * t_{opt}^{slope}$;

        end

        if $\delta_y^{rms} > \epsilon_y^{rms}$

            $t_3 = \mathrm{Optimize\_}f_3(z_y^r, b_y)$;

            $t_{opt}^{slope} = t_{opt}^{slope} + t_3$;

            $t_{opt}^{slope} = t_{opt}^{slope} - \min(t_{opt}) + t_{min}$;

            $z_x^r = z_x^d - b_x * t_{opt}^{slope}$, $z_y^r = z_y^d - b_y * t_{opt}^{slope}$;

        end

    end

end

$\epsilon_x^{rms}, \epsilon_y^{rms}$: specified RMSs of the residual slope errors; i_{max}: the max number of iterations; $\delta_x^{rms}, \delta_y^{rms}$: current RMSs of the residual slope errors; t_{min}: minimum dwell time added to each dwell point.

Table S2 Alternating 3-objective optimization algorithm for the hybrid method. The additional, height-based operations are highlighted.

Input: ϵ^{rms}, ϵ_x^{rms}, ϵ_y^{rms}, z^d, z_x^d, z_y^d
Output: t_{opt}^{hybrid}
Initialization: i_{max}, t_{min}, $z^r = z^d$, $z_x^r = z_x^d$, $z_y^r = z_y^d$, $t_{opt}^{hybrid} = 0$;
while $i = 1$ **to** i_{max} **do**
    $\delta^{rms} = \mathrm{RMS}(z^r)$, $\delta_x^{rms} = \mathrm{RMS}(z_x^r)$, $\delta_y^{rms} = \mathrm{RMS}(z_y^r)$;
    If $\delta^{rms} < \epsilon^{rms}$ **and** $\delta_x^{rms} < \epsilon_x^{rms}$ **and** $\delta_y^{rms} < \epsilon_y^{rms}$
        break;
    else
        if $\delta^{rms} \geq \epsilon^{rms}$
            $t_1 = \text{Optimize\_}f_1(z^r, b)$;
            $t_{opt}^{hybrid} = t_{opt}^{hybrid} + t_1$;
            $t_{opt}^{hybrid} = t_{opt}^{hybrid} - \min(t_{opt}^{hybrid}) + t_{min}$;
            $z^r = z^d - b * t_{opt}^{hybrid}$, $z_x^r = z_x^d - b_x * t_{opt}^{hybrid}$, $z_y^r = z_y^d - b_y * t_{opt}^{hybrid}$;
        end
        if $\delta_x^{rms} \geq \epsilon_x^{rms}$
            $t_2 = \text{Optimize\_}f_2(z_x^r, b_x)$;
            $t_{opt}^{hybrid} = t_{opt}^{hybrid} + t_2$;
            $t_{opt}^{hybrid} = t_{opt}^{hybrid} - \min(t_{opt}^{hybrid}) + t_{min}$;
            $z^r = z^d - b * t_{opt}^{hybrid}$, $z_x^r = z_x^d - b_x * t_{opt}^{hybrid}$, $z_y^r = z_y^d - b_y * t_{opt}^{hybrid}$;
        end
        if $\delta_y^{rms} \geq \epsilon_y^{rms}$
            $t_3 = \text{Optimize\_}f_3(z_y^r, b_y)$;
            $t_{opt}^{hybrid} = t_{opt}^{hybrid} + t_3$;
            $t_{opt}^{hybrid} = t_{opt}^{hybrid} - \min(t_{opt}^{hybrid}) + t_{min}$;
            $z^r = z^d - b * t_{opt}^{hybrid}$, $z_x^r = z_x^d - b_x * t_{opt}^{hybrid}$, $z_y^r = z_y^d - b_y * t_{opt}^{hybrid}$;
        end
    end
end

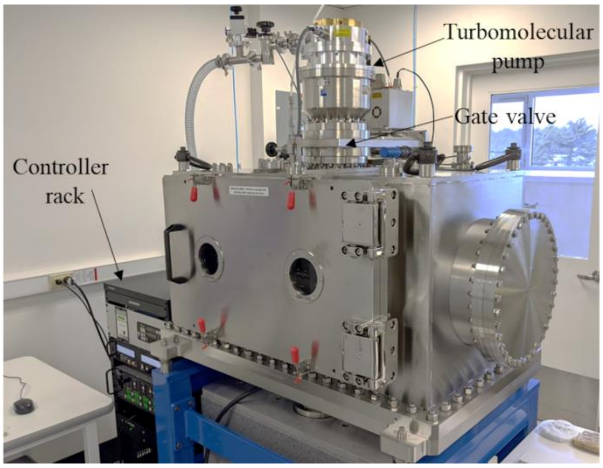

Figure S1 IBF system at NSLS-II. It is equipped with a KDC 10 ion source and an LFN 1000 neutralizer from Kaufman & Robinson Inc.

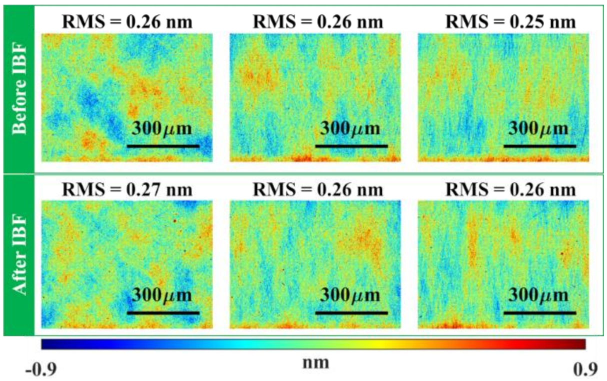

Figure S2 The micro-roughness before and after the IBF process were measured with the Zygo NewView system. The roughness was kept at the sub-0.3 nm RMS level, which demonstrated that the initial surface roughness of the mirror was already below the specification and well-maintained during the IBF process.

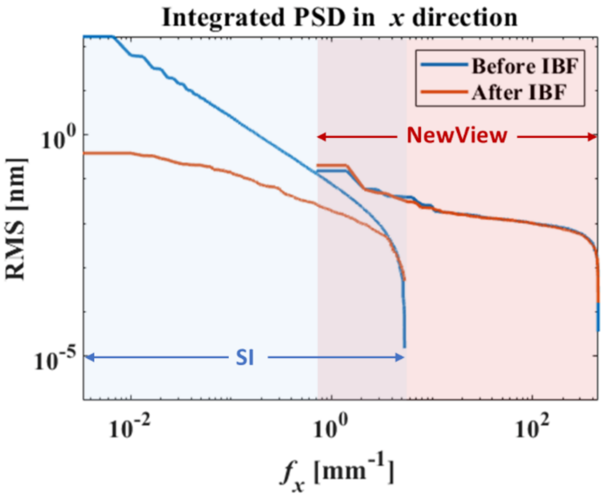

Figure S3 The integrated PSD before and after the IBF process measured with the SI and Zygo NewView systems. It can be observed that the integrated PSDs keep decreasing at spatial frequencies < 5 mm-1 during IBF, indicating that IBF is only capable of correcting the errors in low to middle frequencies and has no effect on the height-frequency components.
